# Effectiveness of Interventions for Meaningful Activity Participation in Homelessness: A Systematic Review

**DOI:** 10.1177/00084174241233519

**Published:** 2024-03-04

**Authors:** Carrie Anne Marshall, Corinna Easton, Elham Javadizadeh, Julia Holmes, Brooke Phillips, Roxanne Isard

**Keywords:** Human activities, Homeless persons, Clinical trials, Intervention research, Poverty, Activités humaines, Études cliniques, Pauvreté, Personnes itinérantes, Recherche interventionnelle

## Abstract

**Background.** Meaningful activity participation has been identified as a key outcome of services designed to support individuals during and following homelessness. Little is known about the effectiveness of interventions for promoting this outcome. **Purpose.** To identify the range and effectiveness of interventions on promoting meaningful activity participation among persons with experiences of homelessness. **Method.** We conducted a systematic review using the Joanna Briggs Institute methodology following PRISMA guidelines including a critical appraisal and narrative synthesis. **Findings.** Of 12,343 titles and abstracts screened, we included 12 studies. The authors of the included studies primarily used standardized measures of meaningful activity engagement. Critical appraisal scores ranged from 50.0 to 77.8. The most common interventions evaluated in the included studies were psychosocial interventions (*n* = 6; 50.0%), followed by case management and housing support interventions (*n* = 4; 33.3%) and Housing First (*n* = 2; 16.7%). While several interventions demonstrated effectiveness in promoting meaningful activity participation including psychosocial and case management interventions, Housing First, Critical Time Intervention, and a peer support intervention were found to be ineffective for promoting engagement in meaningful activity. **Conclusion.** Few intervention studies have been conducted that demonstrate effectiveness for promoting participation in meaningful activity for individuals during and following homelessness. Occupational therapy researchers and practitioners can build on existing evidence by developing and evaluating novel approaches by co-designing interventions in collaboration with persons with experiences of homelessness and service providers.

## Résumé

**Description.** La participation à des activités signifiantes a été identifiée comme un résultat clé des services conçus pour soutenir les individus pendant et après l’itinérance. On sait peu de choses sur l’efficacité des interventions en ce sens. **Objectif.** Identifier la teneur et l’efficacité des interventions visant à promouvoir la participation à des activités signifiantes auprès des personnes ayant une expérience de l’itinérance. **Méthodologie.** Nous avons réalisé une revue systématique recourant à la méthodologie de l’Institut Joanna Briggs selon les lignes directrices PRISMA et comprenant une évaluation critique ainsi qu’une synthèse narrative. **Résultats.** Sur les 12 343 titres et résumés passés en revue, 12 études ont été retenues. Les auteurs des études incluses ont principalement eu recours à des mesures standardisées de l’engagement dans des activités signifiantes. Les scores attribués lors de l’évaluation critique vont de 50,0 à 77,8. Les interventions les plus courantes évaluées dans les études incluses étaient les interventions psychosociales (*n* = 6; 50,0%), suivies par les interventions de gestion de cas et de soutien au logement (*n* = 4; 33,3%) et Logement d’abord (*n* = 2; 16,7%). Si plusieurs interventions se sont révélées efficaces pour promouvoir la participation à des activités signifiantes, notamment les interventions psychosociales et de gestion de cas, Logement d’abord, Critical Time Intervention et une intervention de soutien par les pairs se sont révélées inefficaces pour promouvoir l’engagement dans des activités signifiantes. **Conclusion.** Peu d’études d’intervention démontrent l’efficacité de la promotion de la participation à des activités signifiantes pendant et après l’itinérance. Les chercheurs et les praticiens en ergothérapie peuvent s’appuyer sur les données probantes existantes en développant et en évaluant de nouvelles approches par la coconception d’interventions en collaboration avec des personnes ayant une expérience de l’itinérance et des prestataires de services.

## Introduction

Globally, homelessness is growing. The United Nations has estimated that at least 150 million individuals experience homelessness every year, and a further 1.6 billion people are living in inadequate housing conditions United Nations (2020). As a result, occupational therapists are increasingly supporting individuals who are unhoused or precariously housed in their practice. Not only are there more people who experience homelessness than in recent decades, but occupational therapists are more likely to encounter unhoused and precariously housed persons due to their health profiles, as a large majority of individuals who experience homelessness are living with complex health problems and associated disabilities (Beer et al., 2019; Fazel et al., 2014; Gutwinski et al., 2021). While it has been argued that occupational therapists have been supporting individuals who experience homelessness for decades in traditional roles in community and in-patient mental health settings (Marshall, Gewurtz, et al., 2023; Marshall, Cooke, Gewurtz, Barbic, Roy, Lysaght, et al., 2021), roles dedicated *specifically* to supporting individuals who experience homelessness have only emerged in recent years. To date, there are no known studies that describe how many occupational therapists are currently working in roles with persons who experience homelessness and housing precarity; however, anecdotal evidence indicates that such roles exist and are growing in several countries including Canada, the United States, Ireland, the United Kingdom, Australia, New Zealand and Brazil (Marshall, Cooke, Gewurtz, Barbic, Roy, Ross, et al., 2021).

Alongside the emergence of practice roles in occupational therapy, research aimed at supporting occupational therapy and occupation-based practice has been developing in recent decades, with a range of scoping and systematic reviews that have synthesized research on these topics. These reviews include a comprehensive scoping review of occupation-based practices related to homelessness (Roy et al., 2017), systematic reviews on the effectiveness of occupational therapy interventions during (Thomas et al., 2011) and following (Marshall, Boland, et al., 2021) homelessness, and a systematic review and meta-aggregation of the experiences of occupation during homelessness (Marshall, C. et al., 2019). While these reviews have synthesized research on homelessness from an occupational perspective and identified the range and effectiveness of occupational therapy interventions, none have focused on the effectiveness of interventions aimed at promoting participation in meaningful activities across a range of disciplines. One systematic review exploring the effectiveness of occupational therapy interventions in the transition to housing indicates that occupational therapy interventions that have been developed, evaluated, and reported in existing literature are primarily restricted to evaluating interventions for improving function in one's current daily activities, rather than their ability to promote participation in activities that a person wishes to do but faces barriers to engagement (Marshall, Boland, et al., 2021).

Foundational to the profession of occupational therapy is the belief that participation in meaningful activities is essential for the health and well-being of all humans (Egan & Restall, 2022). In this paper, we define “meaningful activity” as an activity that is subjectively meaningful to the person. A subjective definition is important, as the meaning of an activity can be highly individual and based on a person's ability to perform the activity, their past engagement with the activity, and its cultural significance. Persons who experience homelessness face serious health inequities (Fazel et al., 2014; Gutwinski et al., 2021) that limit access to activities that are meaningful, and existing research associates participation in meaningful activity with increased psychosocial well-being during and following homelessness (Marshall, Cooke, et al., 2024). These barriers include those imposed by the environments in which persons who experience homelessness are situated such as rules in shelters and drop-in centres, a lack of financial resources to facilitate engagement in meaningful activity, and the need to engage in survival activities at the cost of spending time in ways that promote self-actualization (Marshall, Phillips, et al., 2023). As such, finding ways to support access to meaningful activity has been identified as an important goal in supporting individuals as they recover from the trauma of homelessness (Boland et al., 2021; Padgett et al., 2016; Patterson et al., 2015), and is often challenging to achieve (Marshall, C. et al., 2022; Marshall, C. A. et al., 2019). For these reasons, promoting participation in meaningful activity has been recognized by a range of interdisciplinary groups and policymakers as a key outcome of services (Department for Communities and Local Government, 2011; Homeless Hub, 2018; Homeless Link, 2018). Understanding the range and effectiveness of interventions for promoting participation in meaningful activity among individuals who experience homelessness is a critical objective that can inform occupational therapy practice in this area, as well as the practice of other professions that seek to support individuals to attain this outcome.

### The Current Study

Little is known about existing research on the effectiveness of interventions for promoting participation in meaningful activities for individuals who experience homelessness. This information is important for guiding future research, the practice of occupational therapists and other professionals who seek to support individuals to engage in meaningful activity, and to inform policy and funding for programs for persons who experience homelessness. We designed this research to address the research question: What is the range and effectiveness of interventions evaluated in existing peer-reviewed literature on their ability to promote participation in meaningful activity for individuals with experiences of homelessness?

It should be noted that in the current study, we have used the Canadian Definition of Homelessness (Gaetz et al., 2012) to define homelessness. As such, homelessness includes persons who are sheltered, unsheltered, provisionally accommodated, and at risk of homelessness. When we refer to “persons with experiences of homelessness” throughout this paper, we are referring to persons who are currently unhoused, as well as persons who are housed following homelessness.

## Method

We conducted a systematic review of effectiveness studies using the method advanced by the Joanna Briggs Institute (JBI) (Tufanaru et al., 2017) following Preferred Reporting Items for Systematic Reviews and Meta-Analyses (PRISMA) guidelines (Moher et al., 2010). This methodology involves designing a search strategy, conducting a title and abstract screening and full-text review, critical appraisal, and narrative synthesis to describe a body of literature related to the effectiveness reported in existing intervention studies (Aromataris & Munn, 2017). This review was prospectively registered with PROSPERO on October 12, 2021 (Registration# CRD42021290460).

### Search Strategy

We developed a search strategy in collaboration with an academic research librarian and an author on this study (RI). We initially deployed our search in January 2022. This was updated in February 2023. Following PRISMA guidelines (Moher et al., 2010), we searched eight databases that would enable us to locate articles that evaluated the effectiveness of interventions for persons with experiences of homelessness in promoting participation in meaningful activity: ASSIA, CINAHL, EMBASE, Medline, PsychInfo, Social Services Abstracts, Social Work Abstracts and Sociological Abstracts. We translated the search strategies using each database platform's command language, controlled vocabulary, and appropriate search fields using terms related to the concept of homelessness (i.e., homeless*, houseless, unhoused), combined with terms pertaining to meaningful activity (i.e., meaningful activit*, participation, daily activit*) with a Boolean ‘AND.’ In addition to this search, we hand-searched the reference lists of all included articles to identify any additional studies not captured using our search strategy. A sample of our Medline search is provided in Supplementary Appendix 1.

### Study Selection

Acting as two independent raters, several members of our research team (CAM, CE, EJ, JH, BP) conducted a title and abstract screening and full-text review using Covidence, a cloud-based systematic review software program (VeritasHealthInnovation, 2016). We did not include gray literature as we were interested in capturing only moderate to highly rigorous empirical studies that would inform future research on this topic. We included only studies that pertained to broad categories of meaningful activity (i.e., leisure activities, spiritual activities, productivity activities, self-care activities), rather than a specific activity (e.g., healthy eating, participation in a group intervention, prayer) chosen by the study authors. Thus, if a study was specific to one single activity, we did not include it in our review; however, if the study measured the effectiveness of an intervention on a range of meaningful activities, it was included. Furthermore, we did not include interventions that explored the effectiveness of interventions for promoting participation in employment as members of our team have recently published a systematic review which synthesizes this body of literature (Marshall, C. A. et al., 2022). A summary of the inclusion and exclusion criteria used to guide this study is provided in Table 1. Any conflicts emerging at the title and abstract screening and full-text review stages were resolved through discussion and consensus using at least two independent raters. When consensus could not be reached using two raters, a third rater (CAM) was asked to provide a rating to resolve the conflict.

**Table 1.** Inclusion and Exclusion Criteria

Inclusion criteria
Randomized controlled trials (RCT) and quasi-experimental studies Studies aimed at identifying the effectiveness of any intervention on supporting participation in broad areas of meaningful activity (e.g., routine, boredom, meaningful activity engagement, time use, self-care, productivity, leisure, activity balance, social activities, spiritual activities) Studies including persons who are unhoused or housed following homelessness Studies including youth (16–24) and adult (25+) samples All years Studies conducted in high-income countries according to the criteria established by the World Bank (2023)
Exclusion criteria
Non-empirical studies Articles not subjected to peer review Conference abstracts Study protocols Studies focusing on interventions aimed at supporting individuals fleeing natural disaster, war, or conflict or who are situated in refugee camps Studies evaluating interventions aimed at engaging individuals in employment Studies evaluating interventions aimed at increasing participation in specific activities determined by the study authors (e.g., service engagement, volunteering, group participation) Studies evaluating interventions solely for their effectiveness on psychosocial outcomes that do not involve meaningful activity (e.g., mental well-being, community integration, housing tenure) Studies conducted in low- to middle-income countries according to the criteria established by the World Bank (2023) Dissertations and theses Languages other than English

### Critical Appraisal

Four members of our team (CAM, CE, EJ, JH) conducted a critical appraisal of each included study using the JBI Critical Appraisal Checklists for Randomized Controlled Trials (RCT) and Quasi-Experimental Studies (Tufanaru et al., 2017). We assigned a score of one to each criterion rated “yes,” and zero to items rated as “no” or “unclear.” After calculating a summary score, we converted the scores on each rating form to a percentage score between 0 and 100 to facilitate comparison as the total criteria scored on each form differs based on the form used and the study appraised. When a criterion was not applicable to the study that we were appraising, we calculated a percentage based on a reduced number of overall criteria. Scores reaching a minimum of 50.0 were included in this review and narrative synthesis.

### Data Extraction

We created a custom data extraction form in Covidence (VeritasHealthInnovation, 2016) to capture the following information for studies included in our review: intervention name; comparator; participant characteristics (age, gender, race/ethnicity, housing status, 2SLGBTQIA+ status, clinical characteristics); journal discipline; sample country; sample size; study design; critical appraisal score; meaningful activity outcome(s) measured; and findings of the study related to the measure of meaningful activity participation.

### Narrative Synthesis

Using processes described by the JBI (Aromataris & Munn, 2017), we conducted a narrative synthesis of the findings of the included studies. As such, the principal investigator (CAM) arranged each of the included studies into like categories according to intervention type and presented these to other members of the research team. The characteristics of all studies and findings presented in each were then described in detail narratively according to intervention type. We have also provided a narrative summary of strategies used to measure meaningful activity in the included studies.

## Findings

**Figure 1.**
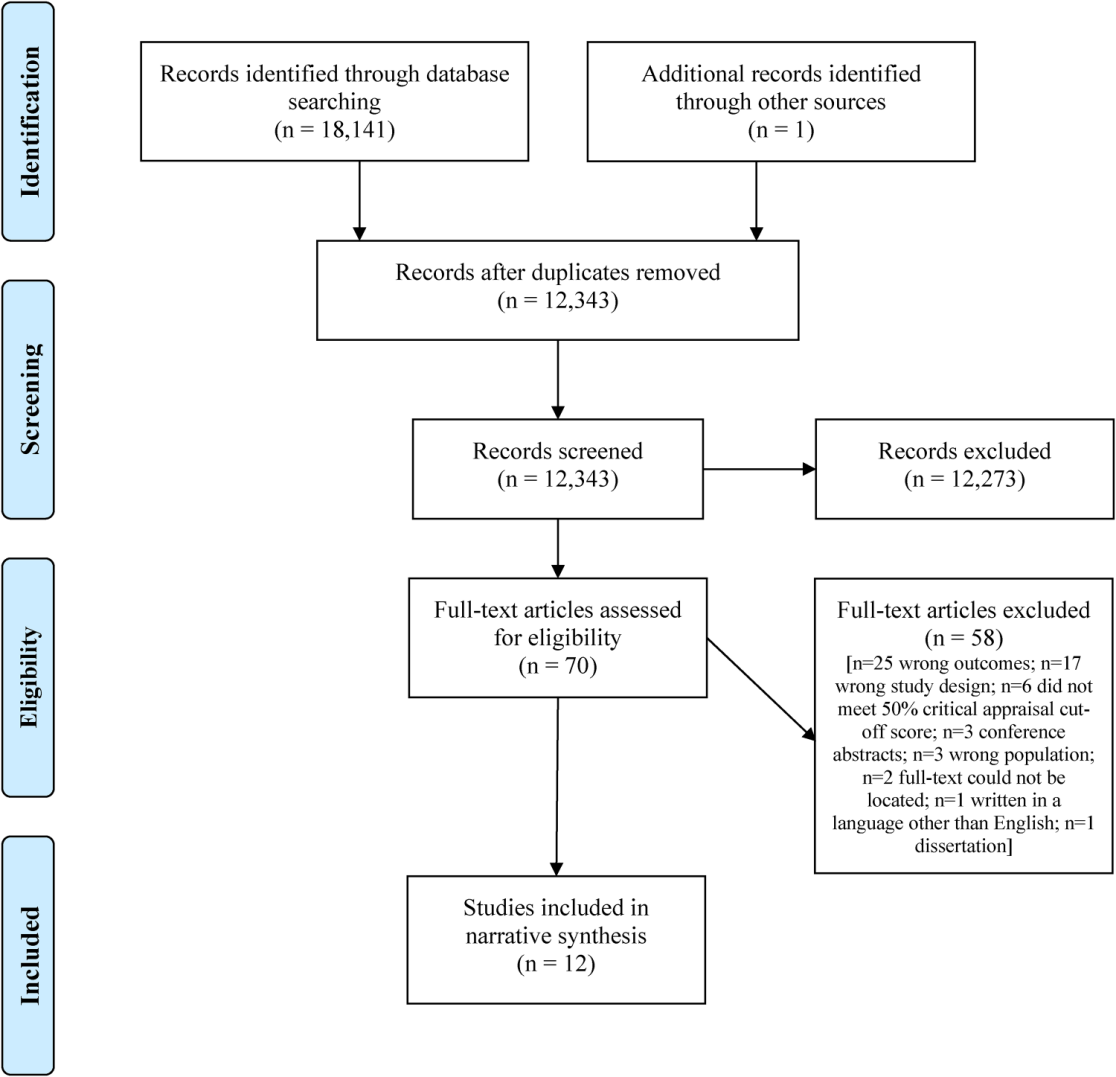
PRISMA flow diagram.

A total of 12,343 titles and abstracts remained following the removal of duplicates, of which 70 were subjected to full-text review. A total of 12 studies were included in our review and narrative synthesis. We did not conduct a meta-analysis as the ways in which the authors of included studies measured meaningful activity engagement were too heterogenous to support a valid and reliable analysis. A summary of the study selection process and reasons for exclusion are provided in a PRISMA flow diagram in Figure 1.

### Study Characteristics

In total, we included 12 studies in our review. Of these, 10 (83.3%) were quasi-experimental studies, and two were RCTs (16.7%). Half of the papers included in this review represented samples in the United States (*n* = 6; 50%), followed by Canada (*n* = 5; 41.7%) and Scotland (*n* = 2; 16.7%). Almost half of the included studies were published in interdisciplinary journals (*n* = 5; 41.7%), followed by psychiatry (*n* = 3; 25.0%), occupational therapy (*n* = 2; 16.7%), and psychology (*n* = 2; 16.7%). A summary of the characteristics of all included studies is provided in Table 2, and a detailed description of the characteristics of individual studies is provided in Table 3.

**Table 2.** Summary of Included Studies (*n* = 12)

Characteristic	
Participant characteristics (*n* = 1824)^a^	*n* (%)
Gender	
Men	1228 (67.3)
Women	208 (11.4)
Other genders	0 (0.0)
Not specified	438 (24.0)
Race/ethnicity^b^	
White	682 (37.4)
Black	444 (24.3)
Hispanic/Latinx	68 (3.7)
Indigenous	48 (2.6)
Mixed race	9 (0.5)
Not specified	618 (33.9)
Sexual orientation	
2SLGBTQIA+	1 (0.05)
Not specified	1823 (99.9)
Clinical characteristics^b^	
Substance use disorder	1089 (59.7)
Physical health condition	408 (22.4)
Psychotic disorder	401 (22.0)
Mood disorder	336 (18.4)
Stress and trauma-related disorder	60 (3.4)
Cognitive health condition	60 (3.4)
Anxiety disorder	20 (1.1)
Concurrent disorder	3 (0.2)
Personality disorder	1 (0.1)
Not specified	230 (12.6)
Housing status	
Housed following homelessness	673 (36.9)
Unhoused	341 (18.7)
Precariously housed	62 (3.4)
Not specified	787 (43.1)
Sample country	No. of studies (%)
United States	6 (50.0)
Canada	5 (41.7)
Scotland	1 (8.3)
Study design	
Quasi-experimental	10 (83.3)
Randomized controlled trial	2 (16.7)
Journal discipline	
Interdisciplinary	5 (41.7)
Psychiatry	3 (25.0)
Occupational therapy	2 (16.7)
Psychology	2 (16.7)
Intervention category	
Psychosocial interventions	6 (50.0)
Case management and housing support	4 (33.3)
Housing First	2 (16.7)

*Note.* Percentage sums do not all equal 100 due to rounding. Due to discrepancies in reporting across individual studies, the number of participants identified in these categories should be treated as estimates.

^a^ Participants in two papers included in this review were from the same study (Collins et al., 2021; Clifasefi et al., 2020). As such, we have only included participants in each of the above categories once to avoid overrepresenting the frequencies of participants in each category.

^b^ Participants frequencies for race/ethnicity and clinical characteristics exceed the total number of participants due to the classification of participants in more than one category in included studies.

**Table 3.** Characteristics of Individual Studies (*n* = 12)

Study	Intervention	Comparator	Participants	Journal discipline	Sample country	Sample size	Study design	Critical appraisal Score (%)	Meaningful activity outcomes measured	Findings
Psychosocial interventions (*n* = 6)										
Alderdice et al. (2022)	The Homeless Occupational Therapy Service (HOTS)	N/A	Age: *M* (*SD*) = 49 (12.6 years) *Gender:* Men (50%) *Race/ethnicity:* ns *Housing status:* Unhoused = 58 (100%) *2SLGBTQIA+ Status:* ns *Clinical characteristics:* All participants presented with physical and/or mental health conditions with 24 (42%) having both. Health conditions ranged from acute (i.e., sprains and fractures) to more chronic health conditions (i.e., substance use, depression, anxiety, diabetes, chronic obstructive pulmonary disease, fibromyalgia, and persistent pain) with chronic conditions being more prevalent. A total of 40 (70%) participants had comorbidities.	Occupational therapy	Scotland	I = 58 C = n/a	Quasi- experimental	50.0	1. Australian Occupational Therapy Outcome Measure Occupational Therapy (AusTOMs-OT) (Unsworth and Duncombe, 2014)	1. Australian Occupational Therapy Outcome Measure Occupational Therapy (AusTOMs-OT) (Unsworth and Duncombe, 2014): Statistically significant improvements were observed in Self-Care (*r* = .55, *p* = .000), Domestic Life-Home (*r* = .54, *p* = .001), and Participation (*r* = .35, *p* = .001) activities.
Boisvert et al. (2008)	Peer Support Community (PSC)	N/A	*Age:* 19–62 years *Gender:* ns *Race/ethnicity:* ns *Housing status:* Housed = 18 (100%) *2SLGBTQIA+ Status:* ns *Clinical characteristics:* substance use disorder = 18 (100%); co-occurring mental illness = 3 (16.7%)	Occupational Therapy	United States	I = 18 C = n/a	Quasi- experimental	71.4	1. Quality of Life Rating scale (QOLRS) (Gust, 1982) 2. The Volitional Questionnaire 4.0 (VQ) (de las Heras et al., 1998)	1. Quality of Life Rating scale (QOLRS) (Gust, 1982): While positive trends were noted on the QOLRS, none reached statistical significance (*p* > .05).^b^ 2. The Volitional Questionnaire 4.0 (VQ) (de las Heras et al., 1998): “No definitive trends could be identified in VQ data” (p. 214).^a^
Clifasefi, Collins & LEAP Advisory Board (2020)	Life-Enhancing Alcohol Management Program (LEAP)	TAU	*Age: M* (*SD*) = 52.66 (8.82 years) *Gender:* Women = 19 (16%) *Race/ethnicity:* White/European American (58%); American Indian/Alaska Native (13%); Black/African American (21%); More than one race (8%); Missing (*n* = 1) *Housing status:* Housed = 116 (100%) *2SLGBTQIA+ Status:* ns *Clinical characteristics:* alcohol use disorder = 66 (100%)	Psychology	United States	I = 66 C = 50	Quasi- experimental	66.7	1. Meaningful activity participation assessment (Eakman et al., 2010)	1. Meaningful activity participation assessment (Eakman et al., 2010): “LEAP participants reported significantly more engagement in meaningful activities than control participants (*p* < .001)” ^b^ (p. 763)
Collins et al. (2021)	Life-Enhancing Alcohol Management Program (LEAP)	N/A [while this study included a control group (see Clifasefi et al., 2020), only findings from the intervention group were included findings reported in this paper]	*Age: M* (*SD*) = 53.67 (7.50 years) *Gender:* Women = 7 *Race/ethnicity:* White/European American = 39 (59%); American Indian/Alaska Native = 11 (17%); Black/African American = 10 (15%); Hispanic/Latinx = 5 (7%); more than one race = 6 (9%) *Housing status:* Housed = 116 (100%) *2SLGBTQIA+ Status:* ns *Clinical characteristics:* alcohol use disorder = 66 (100%)	Psychology	United States	I = 66 C = ns	Quasi- experimental	57.1	1. Participation in meaningful activities offered by LEAP	1. Participation in meaningful activities offered by LEAP: Meaningful activities attended as part of the intervention included: Art hours (*n* = 44; 66.7%); Speaker series (*n* = 28; 42.4%); Poetry (*n* = 24; 36.4%); Talent show (*n* = 22; 33.3%); Zine release party (*n* = 22; 33.3%); Bingo (*n* = 25.8; 17%); Gardening (*n* = 3; 4.5%).
Perreault et al. (2013)	The Urban Breakaway Project	N/A	*Age:* 18–67 years, *M* = 30.3 (*SD* = 12.6) *Gender:* Men = 80% *Race/ethnicity:* ns *Housing status:* Unhoused = 107 (100%) *2SLGBTQIA+ Status:* ns *Clinical characteristics:* ns	Interdisciplinary	Canada	I = 107 C = n/a	Quasi- experimental	55.6	1. Perceived Improvement Questionnaire (PIQ) (Perrault et al., 2010): an adapted version of the PIQ standardized questionnaire was used to evaluate leisure.	1. Perceived Improvement Questionnaire (PIQ) (Perrault et al., 2010): According to the PIQ, participants experienced positive changes related to leisure with 73.8% of participants indicating their activities and hobbies as better or much better than before.
Thulien et al. (2021)	The Identity Project	Delayed intervention group	*Age: M* (*SD*) = 22.9 (2.2 years) *Gender:* Women = 11 (57.9%); Men = 8 (42.1%) *Race/ethnicity:* Black = 7 (36.8%); White = 4 (21.1%); Asian = 4 (21.1%); Other = 4 (21.1%) *Housing status:* Housed = 19 (100%) *2SLGBTQIA+ Status:* ns *Clinical characteristics:* ns	Interdisciplinary	Canada	I = 9 C = 11	Quasi- experimental	77.8	1. Community Integration Scale (Aubry & Myner, 1996)	1. Community Integration Scale (Aubry & Myner, 1996): Participants in the intervention had statistically significant improvements (*p* < .05) and large to very large effect sizes in physical community integration (*d* = 1.79) compared to those in control group. However, the increase in physical community integration immediately post-intervention was sustained until 6 months (*d* = 0.51), after which a small decrease was observed at 9 months (*d* = 0.32), making it no longer statistically significant.
Case management and housing support interventions (*n* = 4)										
Baumgartner & Herman (2012)	Critical Time Intervention (CTI)	TAU	*Age: M* (*SD*) = 38.3 (8.6 years) *Gender:* Men = 71 (75%); Women = 24 (25%) *Race/ethnicity:* African American = 54 (57%); White = 18 (19%); Latino = 17 (18%); Other = 6 (6%) *Housing status:* Not continuously housed = 62 (66%); continuously housed = 32 (34%) *2SLGBTQIA+ Status:* ns *Clinical characteristics:* schizophrenia or schizoaffective disorder = 90 (95%); lifetime substance abuse or dependence = 79 (83%)	Psychiatry	United States	I = 51 C = 44	RCT	61.5	1. Lehman Quality of Life Interview (LQoLI) (social and physical integration subscales) (Lehman, 1988)	1. Lehman Quality of Life Interview (LQoLI) (social and physical integration subscales) (Lehman, 1988): There were no statistically significant differences between the CTI and TAU conditions on social and physical integration measured by the LQoLI scales as reported by the authors.^a^
Rife et al. (1991)	Mobile Case Management	N/A	*Age: M* = 27.8 years *Gender:* Men = 132 (75%); Women = 44 (25%) *Race/ethnicity:* Black = 90 (51.1%); White = 71 (40.3%); Hispanic = 12 (6.8%); Unclassified = 3 (1.8%) *Housing status:* Living in shelters = 79 (44.9%); temporary accommodations = 52 (29.5%); unsheltered = 25 (14.2%); inpatient psychiatric unit = 20 (11.4%) *2SLGBTQIA+ Status:* ns *Clinical characteristics:* mental health needs = 161 (91.5%); substance abuse needs = 68 (38.6%); physical health needs = 47 (26.7%); schizophrenia = 131 (74.4%); other affective disorders or ns = 45	Interdisciplinary	United States	I = 176 C = n/a	Quasi- experimental	66.7	1. Lehman Quality of Life Interview (LQoLI) (Lehman, 1988)	1. Lehman Quality of Life Interview (LQoLI) (Lehman, 1988): After 6 months of the intervention participants reported that their quality of life had significantly improved in leisure activities (*p* <.01).^b^
Tsai et al. (2012)	Collaborative Initiative to Help End Chronic Homelessness (CICH)	N/A	*Age: M* (*SD*) = 45.6 (8.3 years) *Gender:* Men = 413 (76%) *Race/ethnicity:* Black = 269 (49%); White = 204 (37%); Hispanic = 39 (7%); Native American. Asian or Pacific Islander = 33 (6%), missing data = 5 *Housing status:* ns *2SLGBTQIA+ Status:* ns *Clinical characteristics:* major depressive disorder = 157 (28.5%); schizophrenia = 107 (19.5%); bipolar disorder = 105 (19.1%); PTSD = 32 (5.8%); other mental illness = 15 (2.7%); drug abuse or dependence = 295 (53.6%); alcohol abuse or dependence = 290 (52.7%); developmental disability = 60 (10.9%); general medical problem = 361 (65.6%)	Psychiatry	United States	I = 550 C = n/a	Quasi- experimental	66.7	1. Community participation: Participants were asked whether they engaged in 16 common activities (e.g., going to a restaurant or health club, visiting friends) within the past 2 weeks.	1. Community Participation: “Participants also showed a small but statistically significant increase in their community participation over time (*p* <.05). The largest increases were in the number of participants who went to a bank; visited a grocery store; visited close friends, relatives, or neighbors; and went to a shopping centre, mall, or other retail store. However, for nine of the 18 activities (50%), there was no significant change. Across time points, participants reported they engaged in an average of seven of the 16 (44%) community activities” (p. 432).^a,b^
Wasylenki et al. (1993)	Hostel Outreach Program	N/A	*Age: M* = 38 years *Gender:* Men = 34; Women = 25 *Race/ethnicity:* White = 40 (68%); Other races = ns *Housing status:* ns *2SLGBTQIA+ Status:* ns *Clinical characteristics:* schizophrenia = 55 (93%); affective disorder = 2 (3%); personality disorder = 1 (2%); alcohol abuse = 1 (2%)	Psychiatry	Canada	I = 59 C = n/a	Quasi- experimental	77.8	1. Scale for Level of Functioning (SLOF) (Schneider & Struening, 1983)	1. Scale for Level of Functioning (SLOF) (Schneider & Struening, 1983): Activity subscale on the SLOF was significantly lower (a lower score indicates a better level of functioning) across baseline to follow-up (*p* < .001); personal care was significantly lower (*p* < .001); and work was significantly lower (*p* < .003).^b^
Housing First interventions (*n* = 2)										
Cherner et al. (2017)	Housing First (HF)	TAU	*Age:* HF: *M* (*SD*) = 40.6 (9.62 years); TAU: *M* (*SD*) = 40.4 (9.96 years) *Gender:* HF: Men = 40; Women = 49; TAU: Men = 52; Women = 36 *Race/Ethnicity:* ns *Housing Status:* ns *2SLGBTQIA+ Status:* TAU: Transgender = 1 *Clinical characteristics:* depression = 44 (24.7%); PTSD = 28 (15.7%); generalized anxiety disorder = 20 (11.2%); bipolar disorder = 27 (15.2%); schizophrenia/schizoaffective = 18 (10.1%)	Interdisciplinary	Canada	I = 89 C = 89	Quasi- experimental	77.8	1. Multnomah Community Ability Scale (MCAS) (Barker et al., 1994) 2. Lehman Quality of Life Interview (LQoLI) (Lehman, 1988): adapted version of 17 items related to family and social relations, and leisure activities.	1. Multnomah Community Ability Scale (MCAS) (Barker et al., 1994): The TAU condition reported better community functioning on the MCAS at 12-month follow-up than the HF condition (*d* = −0.79, *p* < .001). At 24 months, both HF and TAU conditions demonstrated similar statistically significant improvements in community functioning measured by the MCAS (HF, *d* = −0.57, *p* < .001; TAU *d* = −0.81, *p* <.001). 2. Lehman Quality of Life Interview (LQoLI): There was a significant improvement over time and no main effect of group for leisure activities (pooled *d* = −0.34, *p* < .001) and social relations (pooled *d* = −0.41, *p* < .001) as measured by the LQoLI.
Patterson et al. (2014)	Housing First with Assertive Community Treatment (ACT); Congregate Housing with on-site support (CONG); Housing First with Intensive Case Management (ICM)	TAU	*Age:* At baseline *M* (*SD*) = 40.8 (11.0 years) *Gender:* Men = 73% *Race/ethnicity:* Caucasian = 56%; Other races/ethnicities = ns *Housing status:* Housed = 497 (100%) *2SLGBTQIA+ Status:* ns *Clinical characteristics:* substance dependence = 58%; daily illicit drug use = 25%; severe mental disorders = 73%; less severe mental disorders = 53%	Interdisciplinary	Canada	I = 297 C = 200	RCT	53.8	1. Community Integration Scale (CIS) physical integration subscale (Aubry & Myner, 1996)	1. Community Integration Scale (CIS) physical integration subscale (Aubry & Myner, 1996): There was no significant increase in physical integration over time in the intervention (*p* > .05)

TAU = treatment as usual; I = intervention group; C = control group; ns = not specified.

^a^ *p*-values were not reported.

^b^ Effect sizes not reported.

### Participant Characteristics

Collectively, the included studies represented *n* = 1,824 participants. See Table 2 for a summary of the characteristics of participants in included studies.

### Critical Appraisal

Critical appraisal scores ranged from 50.0 to 77.8 (*M* = 65.2). See Table 3 for scores assigned to each study included in this review.

### Narrative Synthesis

The heterogeneity of outcomes measured in the included studies precluded a meta-analysis. As such, we present our findings narratively.

***Measures of Meaningful Activity Participation.*** Most included studies measured meaningful activity participation using standardized measures. The Lehman Quality of Life Interview (LQoLI) (Lehman, 1988), used in full or in a modified form, was employed in a total of three studies (25.0%) included in this review (Baumgartner & Herman, 2012; Cherner et al., 2017; Rife et al., 1991). This measure explores physical and social integration in the community, which involves spending time engaged in activities that are meaningful, including leisure activities (Lehman, 1988). A total of two studies (16.7%) included in this review (Patterson et al., 2014; Thulien et al., 2021) used the Community Integration Scale (CIS) (Aubry & Myner, 1996). Similar to the LQoLI, the CIS includes a sub-test of physical community integration, which measures meaningful activities in which a person may participate outside of the home such as going to a restaurant or café or participating in sports or recreation (Aubry & Myner, 1996). Other standardized measures of meaningful activity participation used in included studies were the Australian Occupational Therapy Outcome Measure (AusTOMs-OT) (Unsworth & Duncombe, 2014), the Volitional Questionnaire (VQ) (de las Heras et al., 1998), the Quality of Life Rating Scale (QOLRS) (Gust, 1982), the Meaningful Activity Participation Assessment (MAPA) (Eakman et al., 2010), a modified version of the Perceived Improvement Questionnaire (PIQ) (Perreault et al., 2010), the Scale for Level of Functioning (SLOF) (Schneider & Struening, 1983) and the Multnomah Community Ability Scale (MCAS) (Barker et al., 1994).

Non-standardized measures of meaningful activity participation were also used in the included studies. These included the number of activities in which participants engaged of those offered by one intervention called the “Life-Enhancing Alcohol Management Program (LEAP)” (Collins et al., 2021), and a measure of community participation involving questions about engagement in 16 common activities (e.g., going to a restaurant, health club, visiting with friends) (Tsai et al., 2012). See Table 3 for a summary of the measures of meaningful activity participation used by the authors of individual studies.

***Intervention Categories.*** Studies included in this review were clustered according to three intervention categories: (1) psychosocial interventions; (2) case management and housing support interventions; and (3) Housing First (HF) interventions. Each of these categories, the specific interventions that were included in each, and the reported effectiveness on meaningful activity participation reported in individual studies are described in the following narrative.

#### Psychosocial Interventions

Psychosocial interventions were evaluated in a total of six (50%) of the studies included in this review (Alderdice et al., 2022; Boisvert et al., 2008; Clifasefi et al., 2020; Collins et al., 2021; Perreault et al., 2013; Thulien et al., 2021). These interventions included diverse approaches primarily aimed at providing novel services for improving aspects of the mental health and social well-being of individuals with experiences of homelessness. These studies represented a more recent body of literature published between 2008 and 2022. One of these interventions was called the Homeless Occupational Therapy Service (HOTS) (Alderdice et al., 2022), and is described as a community service focused on supporting individuals who are unhoused in shelters or transitional housing who live with mental health and physical disabilities. In this service, an occupational therapist conducts an initial assessment and provides support aimed at facilitating the transition to permanent housing (Alderdice et al., 2022). In their evaluation of HOTS, the authors identified that participating in this intervention resulted in statistically significant improvements in self-care, domestic-home activities, and participating in activities overall. These findings were all associated with moderate to large effect sizes (Alderdice et al., 2022) indicating that this intervention is effective for increasing participation in meaningful activity.

In two papers included in this review, the authors reported on the effectiveness of an intervention called the LEAP, an approach used to support individuals housed following homelessness who were living with alcohol use disorder (Clifasefi et al., 2020; Collins et al., 2021). This intervention was co-designed by residents living in an HF program and involves resident-driven programming aimed at increasing participation in meaningful activities, informed by a harm-reduction approach. In the two papers reporting on this intervention, the authors reported that individuals supported by LEAP participated in a significantly greater number of meaningful activities when compared with participants in a control group (Clifasefi et al., 2020; Collins et al., 2021). In addition, the authors provide descriptive data regarding the activities in which participants supported by LEAP engaged such as art (66.7%), a speaker series (42.4%), poetry (36.4%), a talent show (33.3%), a “zine” release party (33.3%), bingo (17%) and gardening (4.5%). These findings indicate that LEAP may be an important intervention for supporting engagement in meaningful activity following homelessness.

The “Urban Breakaway Project” was designed to provide individuals who are unhoused with the opportunity to participate in a 6-day camp-based retreat in nature, and was evaluated by one of the studies included in this review (Perreault et al., 2013). In this intervention, participants are assigned counsellors who provide guidance and support to participate in activities aimed at developing personal skills, self-esteem, anger management, and problem resolution using a strengths-based approach. The intervention also provides psychoeducation on substance use, attending to basic needs including safety in street contexts, nutrition, and sexual health. Participants are provided with opportunities to engage in sports such as volleyball, canoeing, and other activities (Perreault et al., 2013). The authors of this study indicate that participation in this project resulted in 73.8% of participants reporting improvements in their activities post-intervention, indicating that it shows promise in promoting participation in meaningful activity.

Thulien et al. (2021) evaluated a novel intervention called “The Identity Project,” an intervention involving training youth who have transitioned from homelessness in coaching education as a way of improving “identity capital.” This intervention is composed of a 6-week, six-session intervention aimed at improving a “sense of purpose and control, self-efficacy, and self-esteem” (Thulien et al., 2021, p. 1). This intervention was designed for delivery with youth who often struggle to leave behind their previous homeless identities, attain stability in their housing, and become socially and economically integrated into their community following homelessness. The authors of this study evaluated this intervention in part on its effectiveness for supporting physical community integration (spending time in one's community outside of their apartment) using the CIS (Aubry & Myner, 1996). Their findings indicate that engagement in The Identity Project resulted in statistically significant improvements in physical community integration compared with individuals assigned to a control group, with a large effect size (*d* = 1.79) (Thulien et al., 2021). When conducting follow-up assessments, however, the authors identified that the initial improvement in physical community integration deteriorated over time, with scores on the CIS decreasing to a degree that the increase was no longer statistically significant after 6 months (Thulien et al., 2021). These findings suggest that while participating in the Identity Project was initially effective for improving participation in meaningful activity for youth with histories of homelessness, the effect of this intervention may not have lasting effects over time.

The final intervention included in this category involved a peer support-based program called the “Peer Support Community” (PSC) (Boisvert et al., 2008). This approach focused on supporting individuals living with substance use disorder and histories of homelessness in a permanent supportive housing (PSH) complex to work on abstinence and sustain their tenancies following homelessness. This intervention involved the integration of an occupational therapist and other professional support staff who facilitated the development of a peer community and delivered person-centred programming. Peers both participated in programming and informed the development and delivery of programs (Boisvert et al., 2008). With respect to meaningful activity participation, findings indicate that engagement in the PSC did not contribute to statistically significant improvements on the QOLRS or the VQ (Boisvert et al., 2008). These findings indicate that the PSC was not effective for improving participation in meaningful activity for individuals who participated in this study. See Table 3 for a summary of the findings of each study included in this category, including any reported effect sizes.

#### Case Management and Housing Support Interventions

Case management and housing support interventions were evaluated in a total of four (33.3%) of the studies included in this review (Baumgartner & Herman, 2012; Rife et al., 1991; Tsai et al., 2012; Wasylenki et al., 1993). All of these interventions involved case management services aimed at supporting tenancy sustainment and represent a relatively older body of literature published from 1991 to 2012. One of these studies was an evaluation of an intervention known for its effectiveness in supporting tenancy sustainment following homelessness, called Critical Time Intervention (CTI) (Herman et al., 2011). CTI is a time-limited case management approach in which intensive support is provided in the transition to housing following homelessness (de Vet et al., 2017). In the study included in this review, the authors used the LQoLI (Lehman, 1988) to determine the effectiveness of CTI on social and physical community integration (Baumgartner & Herman, 2012). Findings from this research indicate that involvement in CTI did not improve social or physical community integration for participants in this research, with no statistically significant increases observed on these outcomes (Baumgartner & Herman, 2012). These findings indicate that CTI may not result in improvements in physical community integration (spending time in the community outside of one's apartment) as a measure of participation in meaningful activity.

In another study, the authors evaluated a mobile case management intervention in which case managers met with individuals who were unhoused to provide support throughout the community using an outreach model (Rife et al., 1991). The study authors also used the LQoLI (Lehman, 1988) to evaluate the effectiveness of this intervention on participation in leisure activities, and observed statistically significant improvements on this outcome (Rife et al., 1991). These findings indicate that mobile case management may have a role in improving participation in meaningful activities for individuals who are currently unhoused.

Collaborative Initiative to End Chronic Homelessness (CICH), an approach involving the provision of permanent housing in concert with supportive primary health care and mental health supports was evaluated in one of the included studies (Tsai et al., 2012). This was a large quasi-experimental study in which the authors explored the effectiveness of this intervention on several outcomes including the promotion of “community participation,” measured by self-reported participation in 16 common daily activities. Participants completed interviews across a 1-year period. The authors identified that participants engaged in CICH reported an increase in community participation to a degree that was small, but statistically significant (Tsai et al., 2012), suggesting that CICH may be helpful in supporting participation in meaningful activities for individuals living in PSH following homelessness.

Wasylenki et al. (1993) evaluated the effectiveness of the “Hostel Outreach Program” on participation in meaningful activities. The Hostel Outreach Program comprised case managers providing outreach to unhoused individuals who were receiving support from emergency shelters using an assertive engagement approach (Wasylenki et al., 1993). The authors used the activity subscale of the SLOF (Schneider & Struening, 1983) to measure participation in meaningful activities over an 18-month period. Across this time frame, participants reported a statistically significant increase in meaningful activity participation on the SLOF, with particular improvements reported in personal care (*p* < .001) and work (*p* < .003) (Wasylenki et al., 1993), suggesting that the Hostel Outreach Program may have a role in increasing participation in meaningful activity for individuals who are unhoused. See Table 3 for a summary of the findings of each study included in this category, including any reported effect sizes.

#### Housing First

Two studies (16.7%) included in this review evaluated the effectiveness of HF on improving participation in meaningful activities (Cherner et al., 2017; Patterson et al., 2014). HF is an intervention designed to prevent and end ongoing homelessness through the immediate provision of permanent housing with no preconditions, and wraparound services to support person-centred, recovery-oriented goals (Tsemberis, 2010). This approach is well known for its effectiveness with regard to improving tenancy sustainment following homelessness (Baxter et al., 2019). In one of the included studies, Cherner et al. (2017) found that receiving HF resulted in lower community functioning on the MCAS (Barker et al., 1994) for participants receiving HF when compared with the control group, and no significant differences between the HF and control groups at a 24-month follow-up period. Furthermore, when the HF group was compared with the control group over the course of the study, both groups reported statistically significant increases in the leisure activities and social relations subtests of an adapted version of the LQoLI (Lehman, 1988) over time; however, there were no differences between these groups on this measure (Cherner et al., 2017) indicating that this improvement was a function of time rather than engagement in the HF intervention. In a second study exploring the effectiveness of HF on meaningful activity participation, Patterson et al. (2014) similarly found that there was no statistically significant increase in physical community integration measured using the CIS (Aubry & Myner, 1996) for HF participants. These findings indicate that while HF is effective for promoting tenancy sustainment (Baxter et al., 2019), it may not be effective for promoting engagement in meaningful activity following homelessness.

## Discussion

We conducted this review to identify moderate to high-quality studies that have evaluated interventions on their effectiveness for promoting meaningful activity participation among individuals who experience homelessness. Our findings reveal a small number of studies published by a range of disciplines, including occupational therapy, which have evaluated an array of intervention strategies including psychosocial interventions, case management and housing support interventions, and HF. The included studies were mostly quasi-experimental and represented samples primarily in a North American context (the United States and Canada). The study authors used a range of strategies to measure meaningful activity participation including standardized and non-standardized measures. With regard to effectiveness, most studies provided evidence of improvement in meaningful activity participation with individuals who were housed or unhoused with the exception of HF (Cherner et al., 2017; Patterson et al., 2014), a peer support-based intervention (PSC) (Boisvert et al., 2008) and CTI (Baumgartner & Herman, 2012). The lack of effectiveness of HF, CTI and peer support interventions on meaningful activity engagement, however. does not mean that these approaches are not useful in the support of persons experiencing homelessness. In fact, these approaches have been demonstrated to play a critical role in supporting tenancy sustainment and other aspects of psychosocial well-being beyond meaningful activity engagement in previous research, and as such need to continue to be adopted to target these important outcomes (Baxter, Tweed, Katikireddi & Thomson, 2019; Barker & Maguire, 2017; Munthe-Kaas, Berg & Blaasvaer, 2018). With respect to the outcome of meaningful activity engagement, however, limited number of studies included in this review and the range of interventions evaluated indicate that there is a need for future research on this topic to inform the practice of occupational therapists and other professionals who wish to support persons who experience homelessness to participate in activities that are meaningful in their lives.

### Research and Practice Implications

The limited number of studies included in this review indicates that additional effectiveness studies are needed. All of the included studies evaluated interventions that were broad in scope and did not have a primary purpose of increasing participation in meaningful activity among individuals who experience homelessness. Few existing interventions are known to focus on supporting participation in meaningful activity more generally; however, one intervention called “Action Over Inertia” (AOI), focuses on supporting engagement in meaningful activity among persons living with serious mental illness (Krupa et al., 2008). Research on this approach is limited; however, one pilot study provides initial evidence of the clinical utility of AOI as well as its effectiveness for increasing participation in meaningful activity among persons living with serious mental illness (Edgelow & Krupa, 2011). In other research, qualitative findings indicate that participating in AOI increased awareness of how meaningful activity was related to mental well-being and recovery (Rees et al., 2021). These findings provide evidence indicating some promise in supporting meaningful activity participation and should be evaluated in studies focused on persons with experiences of homelessness in future research.

Given the dearth of interventions designed to promote meaningful activity participation among persons who experience homelessness, researchers and practitioners may consider partnering with persons with lived experience to co-design novel approaches to supporting engagement in meaningful activities during and following homelessness. Bringing together the lived expertise of persons who experience homelessness together with the expertise of practitioners in delivering interventions, and researchers who have knowledge of how to evaluate interventions may be a promising avenue for identifying novel, evidence-based approaches.

### Limitations

While this study represents a comprehensive systematic review of existing research on the effectiveness of interventions on promoting engagement in meaningful activity, we acknowledge some limitations. While the authors of the included studies measured meaningful activity participation as an outcome, none of the included studies were designed primarily to promote participation in meaningful activities. As such, the authors provide little detail regarding the mechanism by which their interventions promoted engagement in meaningful activity, and instead, the findings reflect meaningful activity as one of several measures of psychosocial well-being. The lack of moderate- and high-quality research evaluating interventions designed specifically to increase participation in meaningful activity, however, prevented us from conducting a review of this nature, and our methods were broadened to include all interventions that measured meaningful activity as an outcome during the pilot stage of designing this review. Furthermore, the authors of included studies did not always report use *p*-values or effect sizes in their reporting of outcomes in their studies, and we were limited in our ability to describe the effectiveness of specific interventions due to the lack of statistical detail provided. Researchers who wish to evaluate the effectiveness of interventions for promoting engagement in meaningful activity are encouraged to provide *p*-values and effect sizes in their reporting to enable other researchers and knowledge users to determine the effectiveness of the interventions that have been evaluated.

Other limitations of this review include the lack of diversity of participants in the included studies. The included studies represent primarily White, Black, and heterosexual individuals identifying as men. Researchers may consider conducting studies with more diverse samples in future research. Finally, the findings of this study primarily reflect a North American context, with only one study representing a Scottish sample (Alderdice et al., 2022). Research outside of the United States and Canada is needed to generate a more fulsome understanding of the effectiveness of interventions in other countries and continents. This is essential given the influence of culture on meaningful activity participation (Egan & Restall, 2022).

## Conclusion

Meaningful activity participation is a critical outcome of services designed to support individuals who experience homelessness (Boland et al., 2021; Padgett et al., 2016; Patterson et al., 2015) given its relationship with psychosocial well-being (Marshall, Cooke, et al., 2024; Marshall, C. A. et al., 2019; Marshall et al., 2020). Key organizations and policymakers have identified meaningful activity as a priority in services (Department for Communities and Local Government, 2011; Homeless Hub, 2018; Homeless Link, 2018), yet our findings reveal that only a handful of studies have demonstrated the effectiveness of interventions for promoting this outcome. Research aimed at evaluating promising approaches and designing novel interventions is needed in future research and practice. This research will be vital for informing policy, and the practice of occupational therapists and other practitioners who support individuals during and following homelessness.

## Key Messages

- Few interventions are known to be effective for promoting participation in meaningful activity for persons with experiences of homelessness.
- Interventions that have demonstrated effectiveness include psychosocial and case management approaches.
- The majority of studies exploring the effectiveness of interventions on meaningful activity engagement have been conducted in North America and with samples that lack gender and racial diversity.

## Supplemental Material

sj-docx-1-cjo-10.1177_00084174241233519 - Supplemental material for Effectiveness of Interventions for Meaningful Activity Participation in Homelessness: A Systematic Review Supplemental material, sj-docx-1-cjo-10.1177_00084174241233519 for Effectiveness of Interventions for Meaningful Activity Participation in Homelessness: A Systematic Review by Carrie Anne Marshall, Corinna Easton, Elham Javadizadeh, Julia Holmes, Brooke Phillips and Roxanne Isard in Canadian Journal of Occupational Therapy
