# Supplementary material for: Effectiveness of Interventions for Meaningful Activity Participation in Homelessness: A Systematic Review
Source: Can J Occup Ther. 2024 Mar 4;91(3):256–71. doi: 10.1177/00084174241233519 (PMC11470713; doi:10.1177/00084174241233519)
Supplement: sj-docx-1-cjo-10.1177_00084174241233519 - Supplemental material for Effectiveness of Interventions for Meaningful Activity Participation in Homelessness: A Systematic Review [file sj-docx-1-cjo-10.1177_00084174241233519.docx]

Database: Ovid MEDLINE(R) ALL <1946 to February 11, 2023>

Search Strategy:

--------------------------------------------------------------------------------

1     social connectedness.mp.

2     community affiliation.mp.

3     participation.mp.

4     boring.mp.

5     boredom.mp. or Boredom/

6     Homeless Persons/ or homeless person*.mp.

7     homeless youth.mp. or Homeless Youth/

8     homeless*.mp.

9     houseless.mp.

10     vagran*.mp.

11     "transients and migrants".mp. or "Transients and Migrants"/

12     (transient adj3 (person or persons or people or youth)).mp. [mp=title, abstract, original title, name of substance word, subject heading word, floating sub-heading word, keyword heading word, organism supplementary concept word, protocol supplementary concept word, rare disease supplementary concept word, unique identifier, synonyms]

13     (street adj3 (person or persons or people or youth)).mp. [mp=title, abstract, original title, name of substance word, subject heading word, floating sub-heading word, keyword heading word, organism supplementary concept word, protocol supplementary concept word, rare disease supplementary concept word, unique identifier, synonyms]

14     unhoused.mp.

15     undomiciled.mp.

16     street involved.mp.

17     street involvement.mp.

18     street entrenched.mp.

19     recently housed.mp.

20     precarious housing.mp.

21     unsheltered.mp.

22     rough sleep*.mp.

23     sleeping rough.mp.

24     encampment*.mp.

25     tent city.mp.

26     tent cities.mp.

27     displaced person*.mp.

28     domestic violence.mp. or Domestic Violence/

29     intimate partner violence.mp. or Intimate Partner Violence/

30     fleeing abuse.mp.

31     gender-based violence.mp. or Gender-Based Violence/

32     shelter*.mp.

33     6 or 7 or 8 or 9 or 10 or 11 or 12 or 13 or 14 or 15 or 16 or 17 or 18 or 19 or 20 or 21 or 22 or 23 or 24 or 25 or 26 or 27 or 28 or 29 or 30 or 31 or 32

34     meaningful activit*.mp.

35     meaningful engagement.mp.

36     significant engagement.mp.

37     daily activity.mp.

38     daily activities.mp.

39     "activities of daily living".mp. or "Activities of Daily Living"/

40     IADLs.mp.

41     ADLs.mp.

42     instrumental activities of daily living.mp.

43     meaningful action.mp.

44     important activit*.mp.

45     purposeful activities.mp.

46     purposeful activity.mp.

47     significant activit*.mp.

48     bored.mp.

49     ennui.mp.

50     social integration.mp. or Social Integration/

51     social engagement.mp.

52     social participation.mp. or Social Participation/

53     community participation.mp. or Community Participation/

54     community engagement.mp.

55     time-use.mp.

56     productivity.mp.

57     Volunteers/ or volunteer*.mp.

58     physical activit*.mp.

59     Leisure Activities/ or leisure activit*.mp.

60     recreational activities.mp.

61     Recreation/ or recreation activit*.mp.

62     art-based activity.mp.

63     art-based activities.mp.

64     art based activity.mp.

65     art-based activities.mp.

66     spiritual activity.mp.

67     spiritual activities.mp.

68     religious activities.mp.

69     religious activity.mp.

70     therapeutic activit*.mp.

71     therapeutic engagement.mp.

72     therapeutic participation.mp.

73     activity participation.mp.

74     1 or 2 or 3 or 4 or 5 or 34 or 35 or 36 or 37 or 38 or 39 or 40 or 41 or 42 or 43 or 44 or 45 or 46 or 47 or 48 or 49 or 50 or 51 or 52 or 53 or 54 or 55 or 56 or 57 or 58 or 59 or 60 or 61 or 62 or 63 or 64 or 65 or 66 or 67 or 68 or 69 or 70 or 71 or 72 or 73 or 75

75     leisure.mp.

77     33 and 74

***************************
